# *Shenfu* Injection Promotes Vasodilation by Enhancing eNOS Activity Through the PI3K/Akt Signaling Pathway *In Vitro*

**DOI:** 10.3389/fphar.2020.00121

**Published:** 2020-02-26

**Authors:** Jinqiang Zhu, Wanshan Song, Shixin Xu, Yan Ma, Baoyu Wei, Hongwu Wang, Shengyu Hua

**Affiliations:** ^1^ Institute of Traditional Chinese Medicine, Tianjin University of Traditional Chinese Medicine, Tianjin, China; ^2^ Tianjin State Key Laboratory of Modern Chinese Medicine, Tianjin University of Traditional Chinese Medicine, Tianjin, China; ^3^ Encephalopathy Acupuncture Department, Second Affiliated Hospital of Tianjin University of Traditional Chinese Medicine, Tianjin, China; ^4^ Medical Experiment Center, First Teaching Hospital of Tianjin University of Traditional Chinese Medicine, Tianjin, China; ^5^ Tianjin Key Laboratory of Translational Research of TCM Prescription and Syndrome, First Teaching Hospital of Tianjin University of Traditional Chinese Medicine, Tianjin, China; ^6^ Public Health Science and Engineering College, Tianjin University of Traditional Chinese Medicine, Tianjin, China; ^7^ College of Chinese Medicine, Tianjin University of Traditional Chinese Medicine, Tianjin, China

**Keywords:** *Shenfu* injection, vasodilation, nitric oxide (NO), endothelial nitric oxide synthase (eNOS), PI3K/Akt signaling pathway

## Abstract

Vasomotor dysfunction is one of the key pathological aspects of shock and heart failure (HF). *Shenfu* injection (SFI) has been widely used for the treatment of shock and HF in China. Pharmacological studies have suggested that SFI can reduce peripheral circulation resistance and improve microcirculation. However, whether it has a regulatory effect on macrovascular has not been elucidated. In this study, we used thoracic aorta rings isolated from *Wistar* rats and the human umbilical vein cell line (EA.hy926) to explore the vasodilative activity of SFI and its potential mechanisms. The relaxation due to SFI was measured after pre-treatment with selective soluble guanylate cyclase (sGC) inhibitor or cyclooxygenase (COX) inhibitor and compared with the vasodilation effect of SFI only treated with norepinephrine (NE). The contents of NO, endothelin-1 (ET-1), endothelial nitric oxide synthase (eNOS), COX-1, 6-K-PGF_1α_, and caveolin-1 were evaluated respectively. Additionally, the level of eNOS mRNA and total eNOS and its phosphorylation were studied to investigate the potential mechanisms involved. Experimental results showed that SFI markedly attenuated NE-induced vasoconstriction but that this effect was significantly eliminated after pre-incubation with the selective sGC inhibitor 1-H-[1, 2, 4] oxadiazolo [4, 3-α] quinoxaline-1-one (ODQ), instead of the COX inhibitor indomethacin (INDO). SFI significantly increased the eNOS content and up-regulated the eNOS mRNA expression, while it did not affect the content of COX-1 and 6-K-PGF_1α_. SFI also markedly increased NO content but significantly reduced the content of ET-1 and caveolin-1 in the cell supernatant. Furthermore, it promoted the expression of total eNOS and the phosphorylation of eNOS at serine (Ser) 1177 but inhibited the phosphorylation at threonine (Thr) 495, which was significantly reversed by PI3K-specific inhibitor LY294002. In conclusion, our study showed the vasodilation effect of SFI in thoracic aorta is mediated entirely by enhancing eNOS activity through the PI3K/Akt signaling pathway, providing novel knowledge on the effect of SFI on shock and HF for future clinical applications.

## Introduction

Cardiovascular disease (CVD) is currently the leading cause of death, and around 17.5 million people died of the disease worldwide in 2015, accounting for 31% of deaths in the whole world (Mendis et al., 2015). Although more and more single-component and single-target chemical medicines are being developed, it is still difficult to prevent the increasing rate of morbidity and mortality of CVD; this may be because of serious adverse reactions and high medical costs. Traditional Chinese medicine and its compound may have advantages in treating CVD owing to its multi-component, multi-target, multi-effect features (Dong et al., 2017; Li et al., 2019).

*Shenfu* injection (SFI), a traditional Chinese herbal medicine, is composed of Red Ginseng (the dried root or rootstalk of *Panax ginseng* C. A. Mey) and Fuzi (the lateral root of *Aconitum carmichaeli* Debx) (Zhang, 2016). It is approved by the Chinese State Food and Drug Administration (medicine manufacturing approval number: Z51020664) and has been widely used in the treatment of patients with shock and heart failure (HF) in China (Huang et al., 2019; Wang et al., 2019). The occurrence and development of shock and HF are closely related to vascular activity (Widlansky et al., 2003; Penna et al., 2006; Gulati, 2016). It was reported that SFI could effectively improve microcirculation in shocked rats (Li et al., 2017), improve the microvascular blood flow and coronary perfusion pressure (CPP) during ventricular fibrillation (VF) and cardiopulmonary resuscitation (CPR), and reduce the shocks and duration of CPR (Wu et al., 2016). In addition, SFI can dilate the coronary through promoting NO release (Li et al., 2014) and effectively reduce the damage of vascular endothelial cells (VECs) (Huang et al., 2011).

Endothelial nitric oxide (NO), synthesized by L-arginine catalyzed by endothelial nitric oxide synthase (eNOS) in VECs, was reported to have a significant effect on maintaining the balance of circulation and modulating the vascular tone (Yao et al., 2013). The production of endothelial NO mainly depends on the activity of eNOS. Phosphorylation of eNOS is one of the key factors determining its activity, which is closely related to the phosphatidylinositol 3-kinase/protein kinase B (PI3K/Akt) signaling pathway (Ohkita et al., 2012). Previous studies have shown that vasoactive intestinal polypeptide has a vasorelaxation effect on rat isolated pulmonary artery rings through the PI3K/Akt/eNOS signaling pathway (Zhang et al., 2010), which suggested that this signaling pathway plays a vital role in regulating the vascular activity. Our previous research showed that SFI could dose-dependently inhibit the vasoconstriction induced by potassium chloride (KCl) and norepinephrine (NE) in endothelium-intact thoracic aorta rings but not in the removal of endothelium thoracic aorta rings and that treatment with 1 µM L-NAME eliminated SFI-induced relaxation (Zhu et al., 2013). In the light of the important role of NO in vasomotor function, we aim to evaluate whether the endothelium-dependent vascular relaxation (EDVR) of the thoracic aorta in response to SFI is mediated entirely by NO and whether its mechanism that it promotes eNOS activation through the PI3K/Akt signaling pathway.

## Materials and Methods

### Shenfu Injection

SFI, produced by Ya’an Sanjiu Pharmaceutical Co., Ltd., is composed of Red Ginseng (the dried root or rootstalk of *Panax ginseng* C. A. Mey, 1 mg/ml) and Fuzi (the tuber of *Aconitum carmichaelii* Debeaux, 2 mg/ml). The workflow of SFI preparation is presented in previous research (Liu et al., 2019), and its quality was strictly controlled in compliance with the standard of the China Food and Drug Administration (approval No: WS3-B-3427-98-2013) and ensured by using fingerprint technology during production (Liu et al., 2019). The main active ingredients of SFI are ginsenosides and aconitine. Their chemical structures are shown in the **Supplementary Figure 1** (Wu et al., 2016). Furthermore, 12 ginsenosides and two aconitum alkaloids in 22 batches of SFIs (number of batches: 120609, 110804, 131006010, 131005010, 131013010, 131008010, 130902010, 130813010, 130812010, 130904010, 130905010, 130903010, 130505010, 130506010, 130508010, 130606010,130605010, 130604010, 130713010, 130715010, 130705010, and 130703010, Sichuan, China) were identified by Dr. Yanxu Chang, a member of our team from Tianjin University of Traditional Chinese Medicine (Tianjin, China) and their concentration calculated by the traditional method. The experimental results show that the types and contents of the chemical components in the 22 batches of SFIs were extremely stable. The chemical fingerprint of SFI (number of batch: 110804, Sichuan, China) is shown in the **Supplementary Figure 2**, and the concentrations of the 12 ginsenosides (Re, Rg_1_, Rf, S-Rg_2_, S-Rh_1_, Rb_1_, Rc, Rb_2_, Rb_3_, Rd, S-Rg_3_, and S-Rh_2_) and two aconitum alkaloids (benzoylmesaconine and benzoylhypacoitine) were 60.0 μg/ml, 105μg/ml, 36.6μg/ml, 28.6μg/ml, 15.9μg/ml, 47.6μg/ml, 67.6μg/ml, 53.6μg/ml, 8.60μg/ml, 17.6μg/ml, 22.2μg/ml, 14.5μg/ml, 26.2μg/ml, and 2.24μg/ml, respectively (Ge et al., 2015).

### Aortic Ring Preparation

This study was carried out in strict accordance with the recommendations in the Guidance Suggestions for the Care and Use of Laboratory Animals issued by the Ministry of Science and Technology of China. The protocol was approved by the Laboratory Animal Ethics Committee of Tianjin University of Traditional Chinese Medicine (Permit Number: LAEC2013002). Male *Wistar* rats, 6–8 weeks, weighing 250–300 g, were killed by decapitation. The thoracic aorta was immediately isolated and immersed in an oxygenated Krebs-Henseleit (K-H) solution (in mM: NaCl, 118; KCl, 4.75; MgSO_4_·7H_2_O, 1.2; KH_2_PO_4_, 1.2; CaCl_2_, 2.5; NaHCO_3_, 25; D-glucose, 11) (Xu, 1994) at room temperature. After careful removal of the adipose tissue, it was cut into several 2–3 mm wide segments from the distal and proximal ends (Zhu et al., 2013).

### Measurement of Vascular Relaxation

According to previous experimental methods (Zhu et al., 2013), the aortic rings were suspended between two parallel stainless steel hooks in the organ bath (Radnoti, AD Instruments Pty Ltd., Australia). One hook was fixed, while the other was connected to a force transducer (AD Instruments Pty Ltd., Australia) for the measurement of isometric tension. To keep the blood vessels living, the organ bath was ﬁlled with 10 ml K-H solution, maintained at 37.0°C, pH 7.3–7.4, and bubbled with 95% O_2_ and 5% CO_2_. During the resting periods, the aortic rings were stretched progressively to a basal tension of 2.0 g and allowed to equilibrate for at least 90 min. The organ bath solution was replaced with a pre-warmed and oxygenated K-H solution every 15 min. Thereafter, to standardize the experiments after stabilization at the beginning of the experiments, each aortic ring was ﬁrst repeatedly contracted with 60 mM KCl till the plateau of contractions remained within 5% between two consecutive contractions. After washing with pre-warmed and oxygenated K-H solution three times until muscle tension returned to the basal level, the aortic rings were incubated with α_1_-adrenoceptor agonist norepinephrine (NE, 1 µM) to evoke a steady contraction and then relaxed by acetylcholine (ACh, 10 µM) for assessment of the endothelial function. Rings with less than 60% relaxation in response to ACh were excluded from this experiment. The contractility of vascular rings with endothelium intact was measured isometrically. After establishing a sustained plateau contraction with 1µM NE, different doses (0.l, 1, 2, or 10 μl/ml) of SFI (number of batches: 110804, Sichuan, China) were added cumulatively to induce a concentration-dependent relaxation in rings, and each dosage was added with an interval of 5 min. In order to assess the contribution of relaxation due to NO or PGI_2_, after repeated wash-out and subsequent equilibration for about 45 min, some rings were respectively exposed to 10 µM selective soluble guanylate cyclase (sGC) inhibitor 1-H-[1,2,4]oxadiazolo [4,3-α]quinoxalin-1-one (ODQ) or 10 µM cyclooxygenase (COX) inhibitor indomethacin (INDO) for 20 min prior to application of NE.

### Cell Culture

The vial contained EA.hy 926 cells (ATCC^®^ Catalog No. CRL-2922™, Manassas, VA, USA), which were rapidly thawed within 2 min by gentle agitation in a 37 °C water bath. To avoid the possibility of contamination, the O-ring and cap were kept out of the water. The vial was removed as soon as the contents were thawed and decontaminated by dipping in or spraying with 70% ethanol. The vial contents was transferred to a centrifuge tube containing 9.0 ml complete culture medium (Dulbecco’s Modified Eagle’s Medium (DMEM) with high glucose (HyClone, USA) supplemented with 10% fetal bovine serum (FBS, HyClone, USA) and antibiotics including 1×10^5^ U/L penicillin and 1×10^5^ μg/L streptomycin) and centrifuged at 4 °C and 1000 rpm for 10 min. Cells were resuspended with the complete medium and dispensed into a 25-cm^2^ or 75-cm^2^ culture flask at a density of 10^4^-10^5^ cells/ml, then cultured at 37 °C in a 5% CO_2_ incubator with a renewal of medium every 2–3 days. They were subcultured as soon as they grew into confluent monolayer cells. After discarding the culture medium, the cell layer was briefly rinsed with 0.25% phosphate-buffered saline (PBS, containing 8 g NaCl, 0.2 g KCl, 1.44 g Na_2_HPO_4_•12H_2_O and 0.24 g KH_2_PO_4_ per liter, PH 7.4) to remove all traces of serum, which contains trypsin inhibitor. These cells were digested with an appropriate amount of mixed digestive enzymes of 0.25% Trypsin and 0.02% EDTA (mixed at 1:1) until the cell layer was seen to be dispersed by examining cells under an inverted microscope. Digestion was terminated by the complete medium containing 10% FBS. The following procedures including centrifuge, resuspension, and culture are the same as mentioned above.

### Griess Assay

Nitrites are oxidized metabolites of NO, which was evaluated by the Griess assay kit (Beyotime Biotechnology Co., Ltd., Jiangsu, China) to reflect the content of NO in cell supernatant. EA.hy 926 cells (n = 6 donors) were seeded in a 96-well culture plate and analyzed separately. Upon confluency, the medium was changed to FBS-free DMEM and incubated overnight for synchronization prior to treatment. Cells were separately incubated with 1 μM sildenafil (Sigma, USA) and SFI at three concentrations (10, 20, or 40 μl/ml) for 24 h. The amount of accumulated nitrite derived from NO metabolism was determined according to the kit operating instructions. After incubation at room temperature for 10 min, absorbance was measured at 540 nm as the reference wavelength by a microcontent multifunction microplate reader (Infinite M200, NanoQuant, Switzerland). Nitrite concentrations were calculated using a NaNO_2_ standard curve (0-100 μM in cell culture medium). A nitrite standard reference curve was established for each assay.

### *DAF-FMDA* Fluorescence Indicator

EA.hy 926 cells were seeded at 1×10^6^ cells per well in a six-well culture plate. Upon 90% confluency, cells were synchronized and then treated with 1μM sildenafil (Sigma, USA) and 10, 20, or 40 μl/ml SFI for 24 h. DAF-FMDA fluorescence indicator (Beyotime Biotechnology Co., Ltd., Jiangsu, China) was used to detect the intracellular NO content. According to the kit instructions, the method was as follow:

- *In-situ* loading probe: DAF-FM DA was diluted, with its dilution provided in this kit at a 1:1000 ratio to a final concentration of 5 μM. The cell culture medium was removed, and the appropriate volume of diluted DAF-FMDA was added. Cells were incubated at 37 °C for 20 min and then washed three times with PBS (pH 7.4) to adequately remove the DAF-FM DA that did not enter the cells.
- Detection: The fluorescence intensity was directly observed by laser confocal microscopy or detected by fluorescence spectrophotometer, fluorescence microplate reader, or flow cytometry after collecting cells.
- Parameter setting: The intensity of fluorescence was measured before and after stimulation at a 495 nm excitation wavelength and 515 nm emission wavelength in real-time or at time point or single time point.

### ELISA Assay

EA.hy 926 cells (n = 6 donors) were transferred to a 96-well culture plate. Upon 90% confluency, cells were synchronized and respectively treated with drugs as described above. The cell supernatant from each group of cells was collected, and the eNOS, ET-1, COX-1, 6-K-PGF_1α_, and caveolin-1 contents were measured according to the manufacturer’s instructions (ELISA kit; R&D Systems, USA). Absorbance was detected at 450 nm as the reference wavelength using the microcontent multifunction microplate reader.

### RT-PCR

EA.hy 926 cells were seeded at 1×10^6^ cells per well in a six-well culture plate. Upon 90% confluency, cells were synchronized and respectively treated with 1 μM mevastatin (Sigma, USA) and 5, 10, or 20μl/ml SFI for 24 h. Total RNA was extracted from each sample using the High Pure RNA Isolation kit (Roche, USA) according to the manufacturer’s instructions. RNA samples were subsequently reverse-transcribed to cDNA using SYBR^®^ *Premix* Ex Taq™ Reverse Transcription Reagents (Roche, Switzerland) according to the manufacturer’s instructions, and the resulting cDNA was used as a template for RT-PCR ampliﬁcation. RT-PCR was performed with the ABI^®^ PRISM 7500 Sequence Detection System (Applied Biosystems; Foster City, USA) using SYBR^®^ Green PCR Master Mix reagent kits (Roche, Switzerland) and the speciﬁc primers (**Table 1**, Sangon Biotech (Shanghai) Co., Ltd., Shanghai, China). Relative quantification of gene expression between samples was determined using the 2^-△△CT^ method. All samples were run in triplicate from three independent experiments. The mean crossing threshold (CT) values for both the target and internal control genes in each sample were determined, and the 2^-△△CT^ calculations were performed. The fold change and mean were calculated for each sample (Livak and Schmittgen, 2001).

**Table 1:** Primers of real-time RT-PCR.

Genes	Primer/Probe sequences（5’ to 3’） F, forward; R, reverse
β-actin	F: GGATCAGCAAGCAGGAGTA
	R: GGTGTAACGCAACTAAGTCATAG
eNOS	F: CTCATGGGCACGGTGATG
	R: ACCACGTCATACTCATCCATACAC

### Western Blotting

EA.hy 926 cells were treated according to the protocols mentioned in the RT-PCR study. They were homogenized in ice-cold RIPA lysis buffer containing 1mM PMSF (Beijing Solarbio Science & Technology Co., Ltd, Beijing, China) with several shocks, and lysates were centrifugated at 4°C and 12,000 g for 5 min. We collected and detected the total protein concentration of the supernatants using the BCA assay method (Beijing Solarbio Science & Technology Co., Ltd, Beijing, China). Samples containing 20 μg protein were boiled for 5 min with 5% β-mercaptoethanol and separated on a 10% SDS-polyacrylamide gel by electrophoresis (SDS-PAGE). The protein was transferred to the membrane (immobilon-P polyvinylidene difluoride; Millipore Corp., Bedford, MA, USA) and blocked with 5% skimmed milk for 1–2 h. Primary antibodies against total eNOS (9572S, Cell Signaling Technology, Inc., CST, USA), phosphorylated eNOS at Ser1177 (9571S, Cell Signaling Technology, Inc., CST, USA) and Thr 495 (9574S, Cell Signaling Technology, Inc., CST, USA), and β-actin (4970S, Cell Signaling Technology, Inc., CST, USA) were used for overnight incubation at 4℃. Following three washes (10 min per time) with 0.1% Tween-20 in Tris-buffered saline (TBS), the blots were exposed to horseradish peroxidase-conjugated secondary antibodies for 1.5 h and then washed three times using the method above. An enhanced chemiluminescence detection system (ECL reagent, Amersham Pharmacia Biotech, Buckinghamshire, UK) was used to develop the membranes, and a documentation program (FluorChem, Alpha Innotech Corp., San Leandro, CA, USA) was used for densitometry measurement.

### Statistical Analysis

We employed percentage relaxation relative to pre-contraction levels to NE in the statistical analysis of the relaxant responses. Data are shown as mean ± SD, and statistical significance was estimated by Student’s *t*-test for unpaired observation between two groups or by one-way ANOVA test in comparison to multiple groups. A *p*-value of less than 0.05 was regarded to be significant.

## Results

### The Vasodilation Effect of SFI Involves sGC but not COX-1

**Figure 1:**
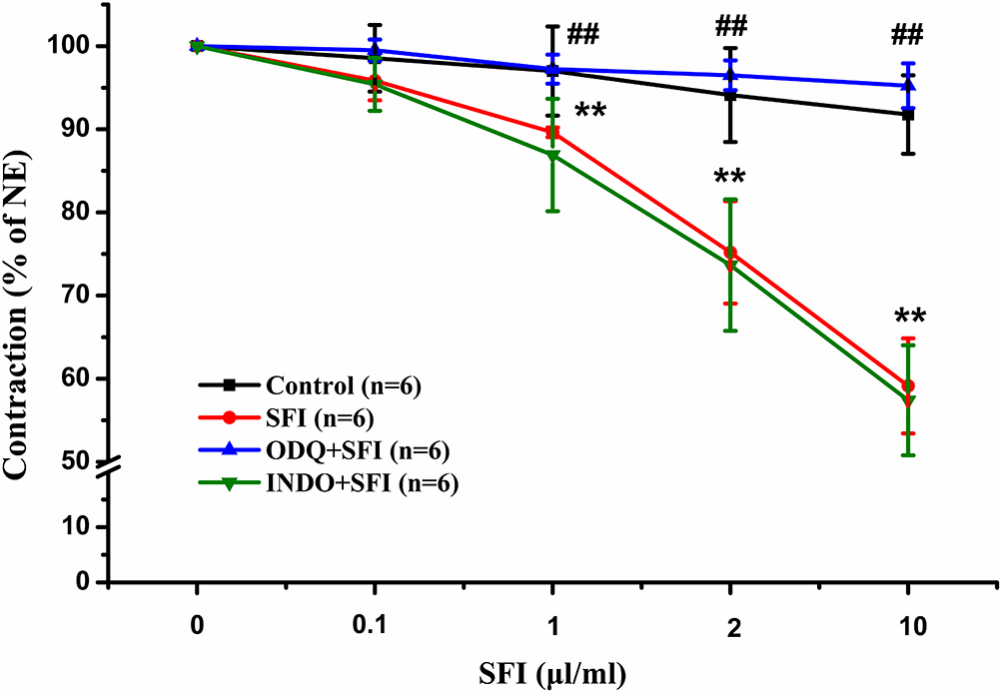
Effects of selective sGC inhibitor ODQ and COX inhibitor INDO on relaxations induced by SFI in isolated thoracic aorta rings contracted with NE. The inhibition of NE (1 μM)-pre-contracted rat thoracic aorta rings with intact endothelium in response to cumulative addition of SFI (0.1, 1, 2 and 10 µl/ml) in the presence and absence of selective sGC inhibitor ODQ (10 µM) and COX inhibitor INDO (10 µM). ODQ, instead of INDO, can obviously restrain the vasorelaxation of SFI. Values are expressed as the mean ± SD, n = 6. ^**^ *P* < 0.01 *vs.* Control (an equal volume K-H solution was added for the control group); ^##^ *P* < 0.01 *vs.* SFI.

To explore the vasodilatation pathways of SFI, we pre-treated the isolated thoracic aorta with 10 µM selective sGC inhibitor ODQ or 10 µM COX inhibitor INDO and compared with the vasodilation effect of SFI only treated with NE. The results showed that SFI (1, 2, and 10 μl/ml) markedly attenuated NE-induced vasoconstriction compared with vasoconstriction in control rat aortas but that this effect was significantly eliminated after pre-incubation with ODQ instead of INDO (**Figure 1**).

### SFI Increases eNOS Content and up-Regulates eNOS mRNA Expression, While it Does not Affect the Prostacyclin Pathway

**Figure 2:**
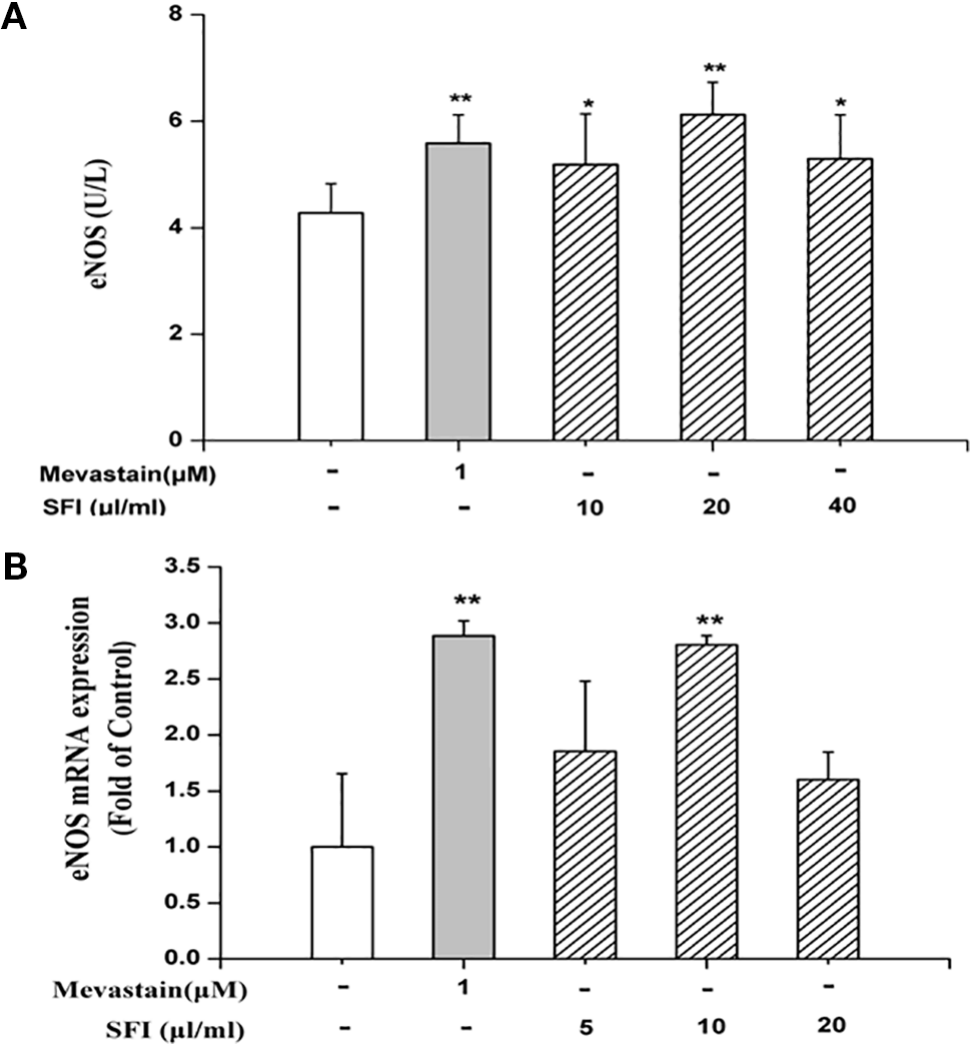
Effect of SFI on eNOS content **(A)** and eNOS mRNA expression **(B)**. 10, 20, and 40 μl/ml of SFI and mevastatin significantly increased eNOS content **(A)**, and 10 μl/ml of SFI obviously up-regulated eNOS mRNA expression, as mevastatin **(B)**. Values are expressed as the mean ± SD, n = 6. ^*^ *P* < 0.05, ^**^ *P* < 0.01 *vs.* Control.

We further explore the vasodilatation pathways of SFI on the molecular biological level. eNOS and COX-1 are the key enzymes in the NO pathway and prostacyclin pathway, respectively. eNOS catalyzes the generation of NO from L-arginine and promotes the conversion of arachidonic acid to prostacyclin (Moncada et al., 1991). PGI_2_, an important vasodilator, is unstable, and it is rapidly degraded into 6-K-PGF_1α_ *in vivo* (Parkington et al., 2004). Thus, we tested the content of 6-K-PGF_1α_ instead of PGI_2_ in the cell supernatant. The results of ELISA showed that 10, 20, and 40 μl/ml of SFI significantly increased eNOS content (**Figure 2A**), and RT-PCR showed that 10 μl/ml of SFI obviously up-regulated eNOS mRNA expression, as mevastatin (**Figure 2B**), while it had no effect on the content of COX-1 and 6-K-PGF_1α_ (**Supplementary Figures 3** and **4**).

### SFI Increases NO Production and Reduces ET-1 Content

**Figure 3:**
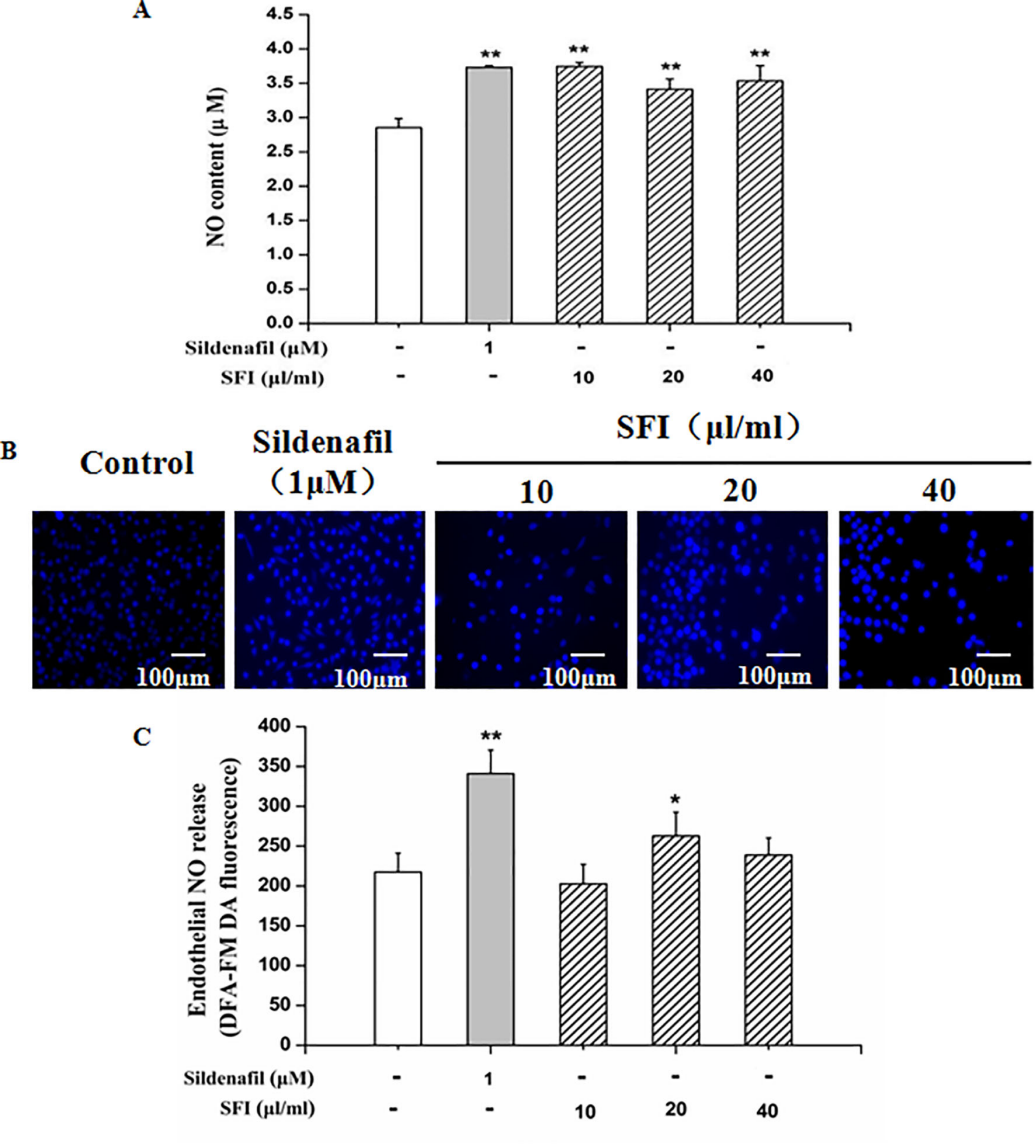
Effect of SFI on extracellular **(A)** and intracellular **(B, C)** NO content. After treatment with different dosages (10, 20, and 40 μl/ml) of SFI for 24 h, all doses of SFI markedly increased NO production in EA.hy 926 cell supernatant **(A)**, and 20μl/ml of SFI also increased the intracellular NO production **(B, C)**, like sildenafil. Values are expressed as the mean ± SD, n = 6. ^*^ *P* < 0.05, ^**^ *P* < 0.01 *vs.* Control.

**Figure 4:**
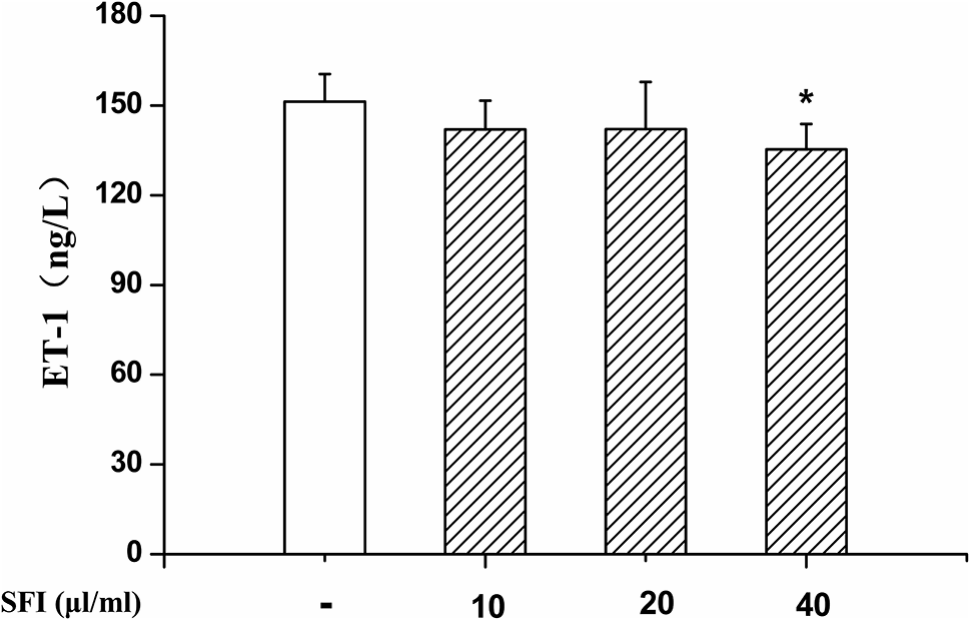
Effect of SFI on ET-1 content. After treatment with different dosages (10, 20, and 40 μl/ml) of SFI for 24 h, 40 μl/ml of SFI significantly reduced ET-1 content in EA.hy 926 cell supernatant. Values are expressed as the mean ± SD, n = 6. ^*^ *P* < 0.05 *vs.* Control.

NO is the strongest diastolic vascular factor, and ET-1 is the strongest vasoconstrictor factor. We tested NO content in EA.hy 926 cells supernatant using Griess assay and in EA.hy 926 cells by DAF-FMDA fluorescence indicator, respectively, and detected the ET-1 content in EA.hy 926 cell supernatant by ELISA. After treatment with different dosages (10, 20, and 40 μl/ml) of SFI for 24 h, all doses of SFI markedly increased NO production in EA.hy 926 cell supernatant (**Figure 3A**), and 20μl/ml of SFI also increased the intracellular NO production (**Figures 3B, C**), like sildenafil, while 40μl/ml of SFI significantly reduced ET-1 content in EA.hy 926 cell supernatant (**Figure 4**).

### Effects of SFI on the Phosphorylation Level of eNOS in the Endothelial Cells

**Figure 5:**
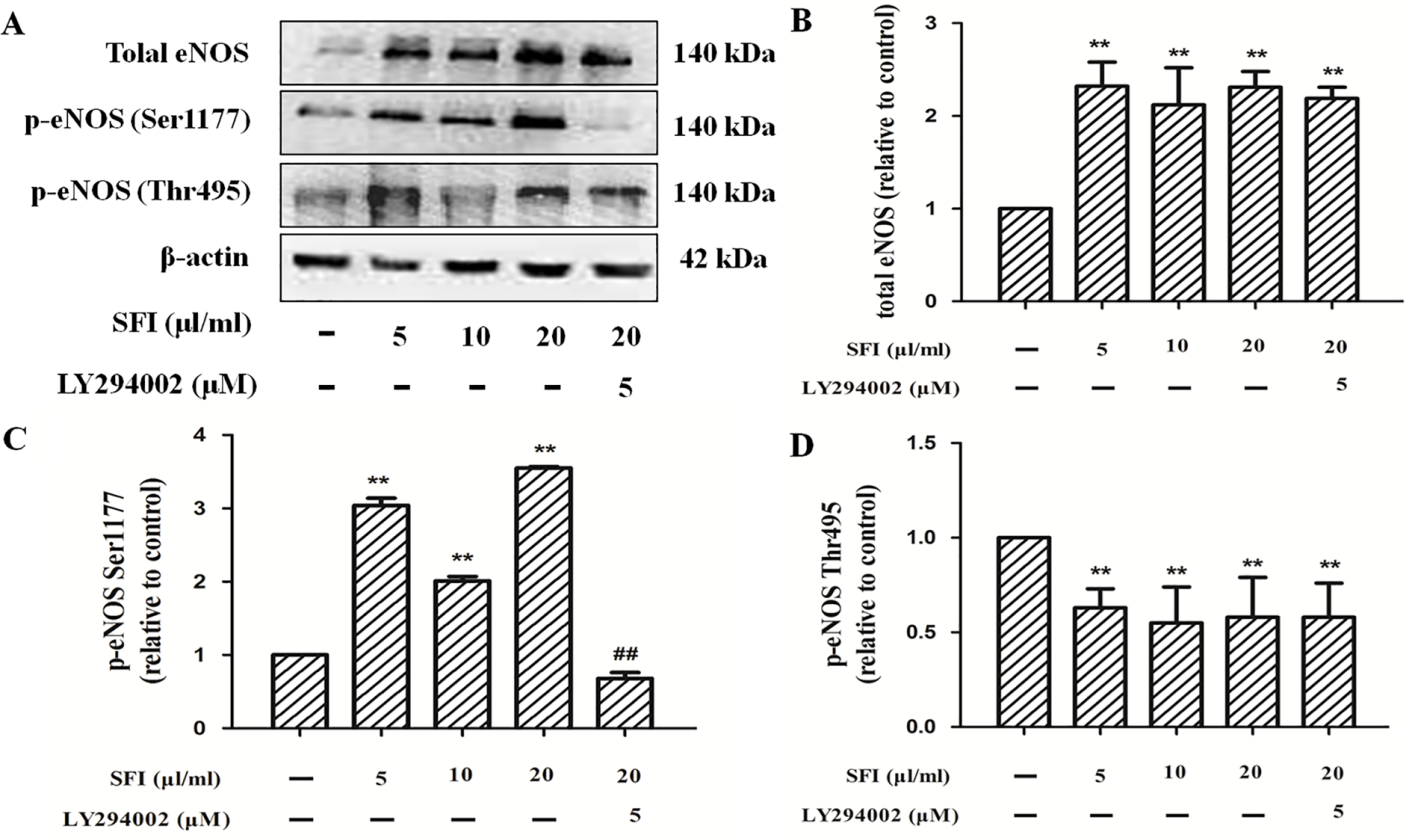
Effect of SFI on the expression of total eNOS and the phosphorylation of eNOS at Ser1177 and Thr 495. Representative blot images demonstrating total eNOS, p-eNOS Ser 1177, p-eNOS Thr 495, and β-actin protein expression levels **(A)**. SFI promoted the expression of total eNOS **(B)** and the phosphorylation of eNOS at Ser1177 **(C)** but inhibited the phosphorylation at Thr495 **(D)**, which was significantly reversed by PI3K-specific inhibitor LY294002. Values are expressed as the mean ± SD, n = 3. ^**^ *P* < 0.01 *vs.* Control; ^##^ *P* < 0.01 *vs.* SFI (20 μl/ml).

Phosphorylation of protein residues is a major influencing factor on eNOS activity. Previous research shows that the phosphorylation of eNOS at Ser1177 can increase its activity. In contrast, the phosphorylation of eNOS at Thr495 can decrease its activity (Dimmeler et al., 1999; Fleming et al., 2001). The results of Western blotting showed that, compared with control, SFI promoted the expression of total eNOS (**Figures 5A, B**) and the phosphorylation of eNOS at Ser1177 (**Figures 5A, C**) but inhibited the phosphorylation at Thr495 (**Figures 5A, D**), which was significantly reversed by PI3K-specific inhibitor LY294002.

### SFI Down-Regulates the Protein Expression of Caveolin-1

**Figure 6:**
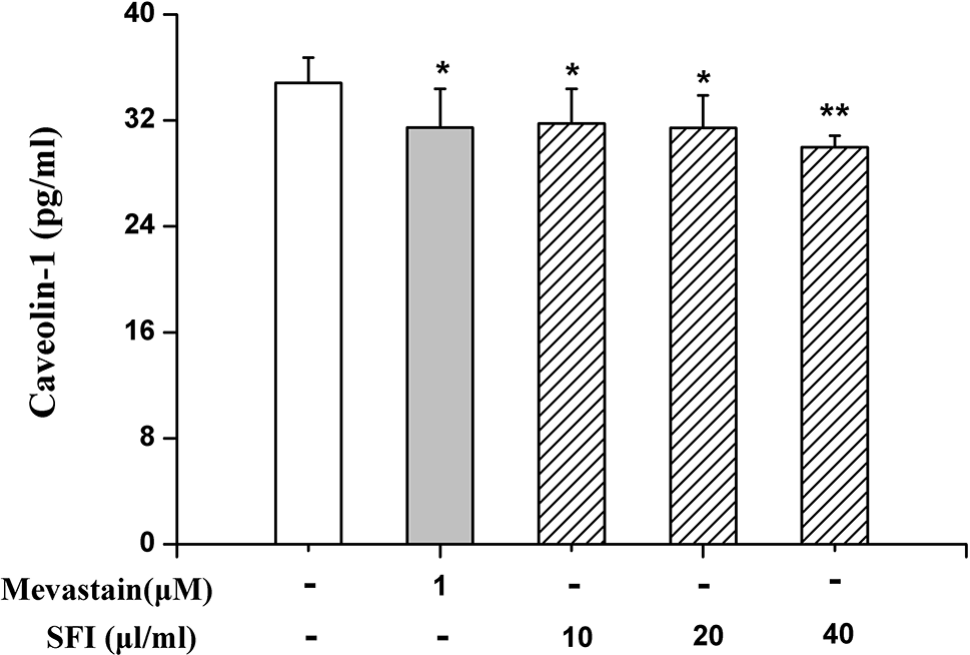
Effect of SFI on the caveolin-1 content in the cell supernatant. SFI significantly reduced the caveolin-1 content in the cell supernatant, like mevastatin. Values are expressed as the mean ± SD, n = 6. ^*^ *P* < 0.05, ^**^ *P* < 0.01 *vs.* Control.

eNOS combined with caveolin-1 can inhibit its activity (Bucci et al., 2000). To preliminarily clarify the mechanism by which SFI regulates eNOS activity, we also measured the caveolin-1 content in the cell supernatant. The results showed that SFI significantly reduced the caveolin-1 content in the cell supernatant, like mevastatin (**Figure 6**).

## Discussion

To our best knowledge, this is the first systematic study of the mechanism behind the vasorelaxation effect of SFI. This study shows that SFI can exert endothelium-dependent vasodilatory effects through the NO-cGMP pathway on the vascular endothelium. Its mechanism is to up-regulate the expression of eNOS mRNA and protein, promote endothelial NO synthesis and release, reduce Cav-1 expression and ET-1 content, promote the phosphorylation of eNOS at Ser1177, and inhibit the phosphorylation at Thr495, resulting in eNOS activation through the PI3K/Akt signal pathway.

Endothelial NO is generated in the VECs by L-arginine (L-Arg) catalyzed by eNOS and plays an important role in relaxing vascular smooth muscle, inhibiting smooth muscle cell proliferation and platelet adhesion and aggregation, which is important for maintaining the circulatory system and the internal environment (Cui et al., 2006; Jia et al., 2006). ET-1 is currently the most known vasoconstrictor substance (Nar et al., 2013). NO and ET-1 produced in VECs are the most important vasoconstrictor substances, and they are among the most important indicators reflecting the function of blood vessels. The dynamic balance between them is an important condition for the normal tension of blood vessels (Theodorakis et al., 2015).

When HF occurs, insufficient cardiac output leads to excessive activation of the sympathetic nervous system and rennin-angiotensin system and endothelial dysfunction, which manifests as a marked increase in vasoconstrictor substances, a marked decrease in vasodilator substances, obvious damage to endothelium-dependent vasodilation, and abnormal contraction of blood vessels. All of this leads to increased systemic circulation and pulmonary circulation resistance, water and sodium retention, and increased cardiac preload and cardiac after-load; at the same time, endothelial dysfunction increases peripheral resistance and cardiac after-load through the peripheral effect (Widlansky et al., 2003; Penna et al., 2006). During the compensatory phase of shock, under the influence of shock factors, EDRF/NO secreted in VECs increases, which exerts a protective effect on systemic tissues and organs; during the decompensation period of shock, EDRF/NO continues to be excessive, and the steady state between vasoactive substances is destroyed, which guides, facilitates, or synergizes with vasodilators, such as adrenomedullin, and then causes an enhancement of vascular over-dilation and cell damage, which forms a vicious cycle, leading to irreversible shock (Wang et al., 2000). Therefore, vasomotor function plays an important role in the pathological process of shock and HF.

The regulation of vasomotor functions is mainly through NO-cGMP and PGI_2_ pathways. The NO synthesized in VECs rapidly enters vascular smooth muscle cells (VSMCs) and acts on soluble guanylate cyclase (sGC) to convert guanosine triphosphate (GTP) to cyclic guanosine monophosphate (cGMP), then activates cGMP-dependent proteases, which causes the dephosphorylation of the myosin light chain, decreases intracellular free Ca^2+^ levels, and reduces the binding of contractile proteins to Ca^2+^, resulting in smooth muscle relaxation and vasodilation (Moncada et al., 1991). PGI_2_ is generated in the VECs by arachidonic acid under the action of COX and prostacyclin synthase and binds to the tissue-specific G protein-coupled receptor PGI_2_ receptor on the cell membrane, which can activate adenylate cyclase, increase the content of cyclic adenosine monophosphate (cAMP), activate protein kinase A (PKA), promote the opening of K^+^ channels, and thus exert vasodilation (Parkington et al., 2004; Fetalvero et al., 2006). PGI_2_ is extremely unstable in aqueous solution and readily hydrolyzes to 6-K-PGF_1_. Our previous studies suggested that SFI has NO-related endothelium-dependent vasodilatory effects (Zhu et al., 2013). This study used an isolated vascular ring experimental method to further clarify the pathway of its vasodilation. The results show that the sGC inhibitor ODQ can significantly eliminate the vasodilation of SFI, while the COX inhibitor INDO has no such effect, which suggested that the endothelium-dependent vasodilation of SFI is related to the NO-cGMP pathway. Moreover, the results of the *in vitro* cell experiments in this study show that SFI can significantly increase the expression of eNOS but that it has no significant effect on the content of COX-1 and PGI_2_ metabolite 6-K-PGF_1_, further verifying that the vasodilation effect of SFI is through the NO-cGMP pathway. On this basis, we investigated the effect of SFI on intracellular and extracellular NO content and extracellular ET-1 content. The results showed that SFI significantly increased NO synthesis and release and decreased ET-1 content, suggesting that SFI can modulate the balance of NO/ET-1, thereby effectively improving vasomotor function.

**Figure 7:**
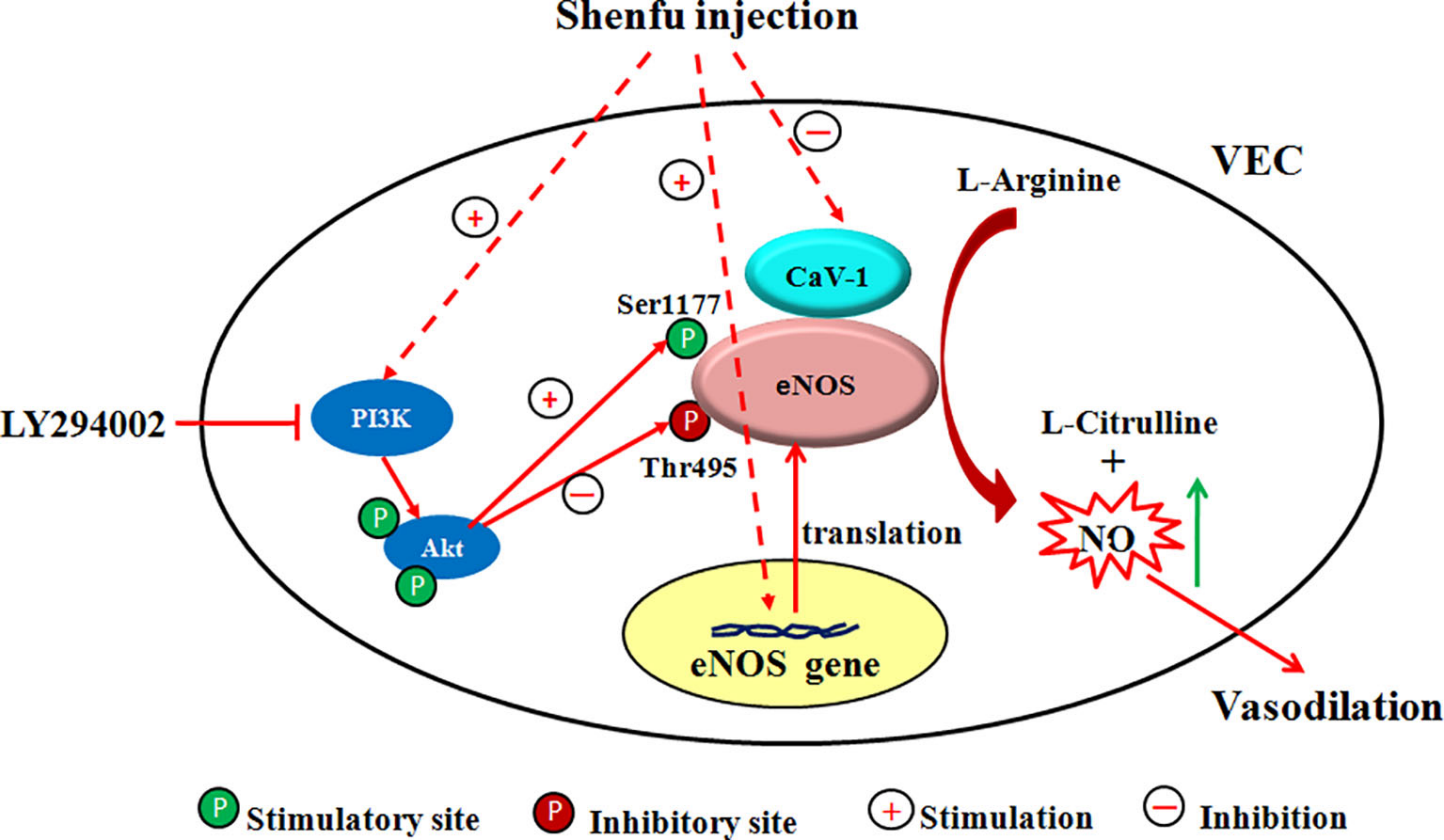
Scheme of the proposed mechanism by which SFI increases eNOS activity. Administration of SFI results in an increase in the gene and protein expression of eNOS and promotes the eNOS phosphorylation at Ser1177 but inhibits the eNOS phosphorylation at Thr 495 and decreases the Cav-1 content, ultimately resulting in enhanced NO production. This effect was antagonized by the PI3K/Akt signaling pathway inhibitor LY294002. The inhibitor used and its location of action is also indicated.

Since the half-life of NO is very short (about 6–30 s), the NO production in the endothelium mainly depends on the activity of the key enzyme eNOS (Laspas et al., 2014). The regulation mechanism of eNOS activity is extremely complex. i) The expression of eNOS mRNA directly affects the amount of eNOS synthesis and thus regulates eNOS activity. ii) The binding of CaM to eNOS enhances the activity of eNOS; conversely, the combination of Cav-1 and eNOS decreases the activity of eNOS (Feron et al., 1996; Garcia-Cardeña et al., 1997; Ghosh et al., 1998). iii) eNOS is readily phosphorylated at serine, threonine, and tyrosine residues. Phosphorylation at Thr495 reduces the binding of CaM to eNOS, thereby inhibiting the activity of eNOS (Fleming et al., 2001), while phosphorylation at Ser1177 can enhance the catalytic ability of eNOS by inhibiting the separation of CaM from eNOS and enhancing the internal electron transfer rate of eNOS (Gallis et al., 1999; McCabe et al., 2000). Akt (i.e., PKB) is an important determinant of phosphorylation at the eNOS Ser 1177, which is involved in the basic activation of eNOS and agonist-induced activation (Fulton et al., 1999; Bauer et al., 2003). It is mainly present in the cytoplasm in an inactive form and must translocate to the cell membrane to activate itself and phosphorylate eNOS (Gonzalez et al., 2002). In addition, it is directly controlled by the PI3K-dependent phosphorylation pathway, and PI3K recruits Akt to the cell membrane, thereby phosphorylating it (Vanhaesebroeck et al., 1997). To investigate the mechanism by which SFI regulates eNOS activity, we used EA.hy926, a fusion of primary cultured human umbilical vein cells with thioguanine-resistant A549 clone under PEG stress, to detect the gene and protein expression of eNOS, the protein expression of Cav-1, total eNOS, and the phosphorylation of eNOS at Ser1177 and Thr495. The results showed that SFI significantly up-regulated the gene and protein expression of eNOS, decreased the Cav-1 content in the cell supernatant, and promoted the phosphorylation of eNOS at Ser 1177 but inhibited the phosphorylation of eNOS at Thr495, thereby enhancing eNOS activity. This effect was antagonized by the PI3K/Akt signaling pathway inhibitor LY294002, which suggested that SFI may promote the phosphorylation of eNOS through the PI3K/Akt signaling pathway (**Figure 7**).

Although this study preliminarily revealed that the mechanism behind SFI’s endothelium-dependent vasodilatation is that it enhances eNOS activity through the PI3K/Akt signaling pathway *in vitro*, the mechanism of eNOS activation is extremely complex and can be divided into gene regulation (regulation of promoter and mRNA stability) and protein regulation (eNOS intracellular translocation, eNOS complex formation, and the phosphorylation of eNOS amino acid residue) (Dias et al., 2011; Zhu et al., 2015). Various regulatory mechanisms are interconnected to form a complex network structure. Therefore, more comprehensive and systematic research on other regulatory mechanisms is needed in the future to fully elucidate the mechanism by which SFI regulates eNOS activity.

It is well-known that vasomotor function is not only related to vascular endothelium but also requires co-regulation of vascular smooth muscle (Dias et al., 2011). Therefore, it is necessary to explore the effect of SFI on signal transduction between VECs and VSMCs in the future to clarify its mechanism of diastolic vasodilation further.

## Conclusion

Our study revealed for the first time that endothelium-dependent vasodilatation effect of SFI occurs through the NO-cGMP pathway but not the PGI_2_ pathway. Moreover, we found that the vasorelaxation effect of SFI is mediated through the PI3K/Akt/eNOS/NO signaling pathway in VECs. Overall, this study provides a scientific basis for the treatment of shock and HF with SFI and lays a foundation for the expansion of its clinical application and secondary development.

## Data Availability Statement

All datasets generated for this study are included in the article/**Supplementary Material**.

## Ethics Statement

This study was carried out in strict accordance with the recommendations in the Guidance Suggestions for the Care and Use of Laboratory Animals issued by the Ministry of Science and Technology of China. The protocol was approved by the Laboratory Animal Ethics Committee of Tianjin University of Traditional Chinese Medicine (Permit Number: LAEC2013002).

## Author Contributions

JZ and BW carried out the experiments. JZ, WS, SX, YM, and HW performed analysis and interpretation of the data. JZ and SH designed the experiments. JZ wrote and revised the manuscript. All of the authors gave final approval to the work.

## Funding

We are grateful for the financial support from the National Natural Science Foundation of China (No. 81973626, 81473381) and the Training Project of Tianjin Institution “Innovation Team” (TD13-5049).

## Conflict of Interest

The authors declare that the research was conducted in the absence of any commercial or financial relationships that could be construed as a potential conflict of interest.
