# Supplementary material for: Shenfu Injection Promotes Vasodilation by Enhancing eNOS Activity Through the PI3K/Akt Signaling Pathway In Vitro
Source: Front Pharmacol. 2020 Feb 26;11:121. doi: 10.3389/fphar.2020.00121 (PMC7054240; doi:10.3389/fphar.2020.00121)
Supplement: Supplementary file 1 [file DataSheet_1.zip › the supplementary materials/the supplementary materials.docx]

**Supplementary materials**

**

Fig. S1 Chemical structures of Ginsenoside and Aconitine. The mainactive ingredients of SFI are ginsenosides and aconitine (Wu et al., 2016).

Fig. S2 Representative chromatograms for simultaneous quantification of the 14 active compounds in Shenfu injection: (A) mixed standards at 203 nm; (B) mixed standards at 235 nm; (C) Shenfu injection sample at 203 nm; and (D) Shenfu injection sample at 235 nm. Peak 1 represents Re, peak 2 Rg_1_, peak 3 Rf, peak 4 S-Rg_2_, peak 5 S-Rh_1_, peak 6 Rb_1_, peak 7 Rc, peak 8 Rb_2_,peak 9 Rb_3_, peak 10 Rd, peak 11 S-Rg_3_, peak 12 S-Rh_2_, peak 13 benzoylmesaconine, and peak 14 benzoylhypacoitine (A. H. Ge et al., 2015).


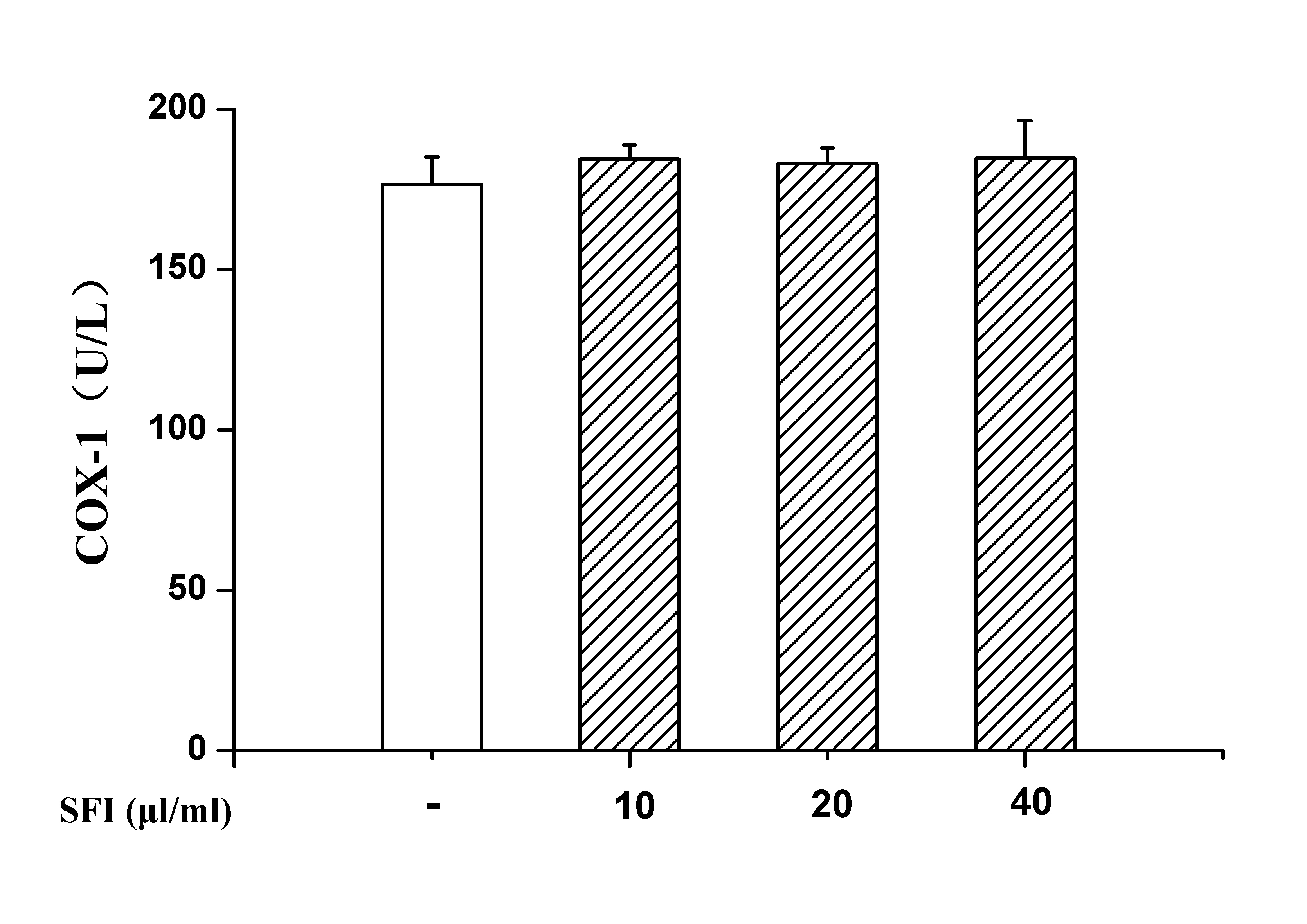


Fig. S3 Effect of SFI on COX-1 content. SFI has no effect on the COX-1 content in the cell supernatant. Values are expressed as the mean±SD, n=6.


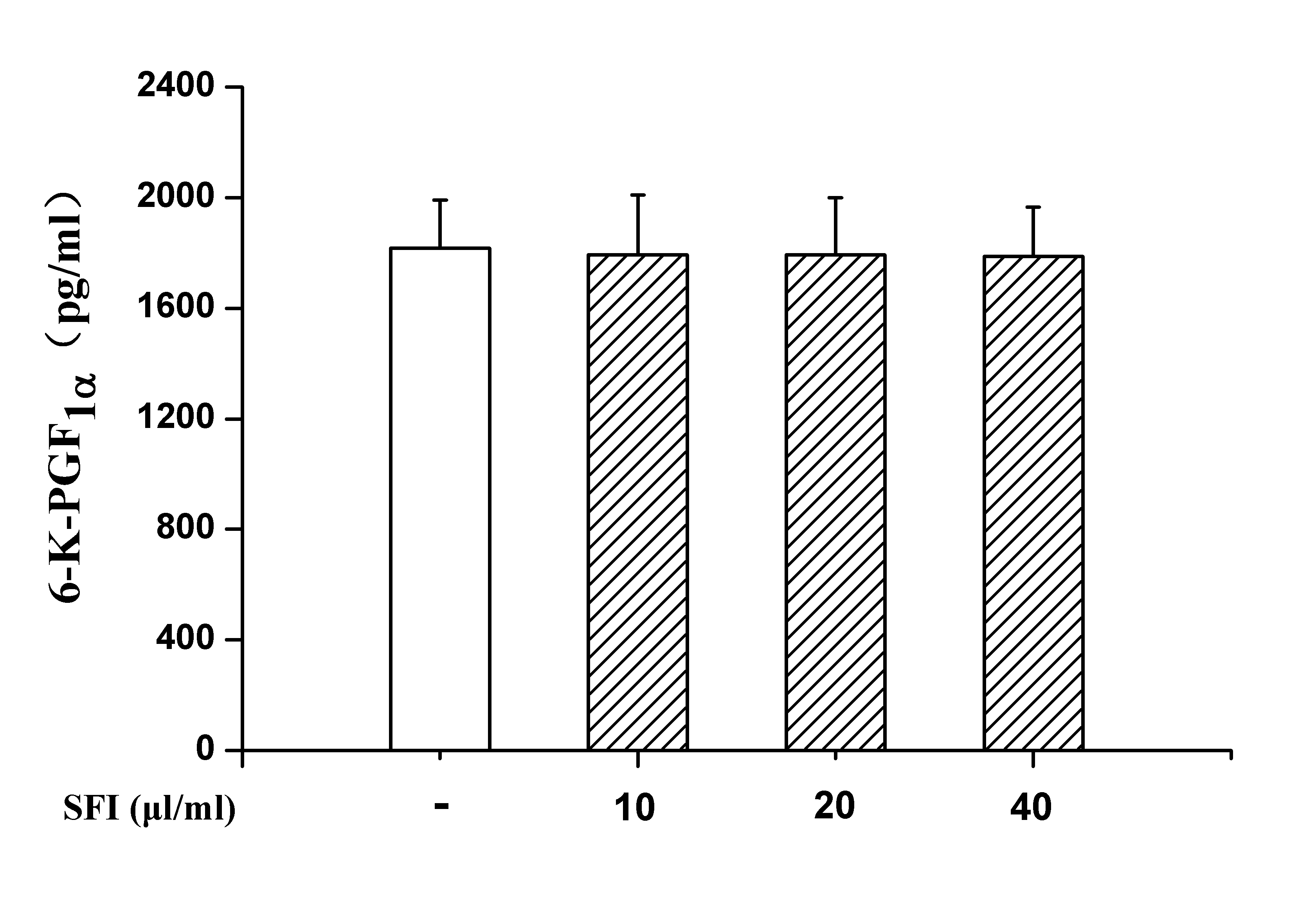


Fig. S4 Effect of SFI on 6-K-PGF_1α_ content. SFI has no effect on the 6-K-PGF_1α_ content in the cell supernatant. Values are expressed as the mean±SD, n=6.
