# Supplementary figures and images for: Shenfu Injection Promotes Vasodilation by Enhancing eNOS Activity Through the PI3K/Akt Signaling Pathway In Vitro
Source: Front Pharmacol. 2020 Feb 26;11:121. doi: 10.3389/fphar.2020.00121 (PMC7054240; doi:10.3389/fphar.2020.00121)

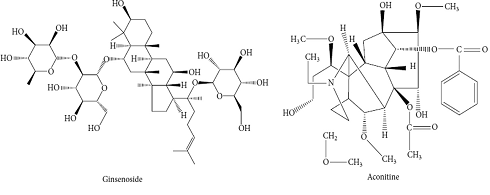

Supplement: Supplementary file 1 [file DataSheet_1.zip › the supplementary materials/Fig. S1.tif]

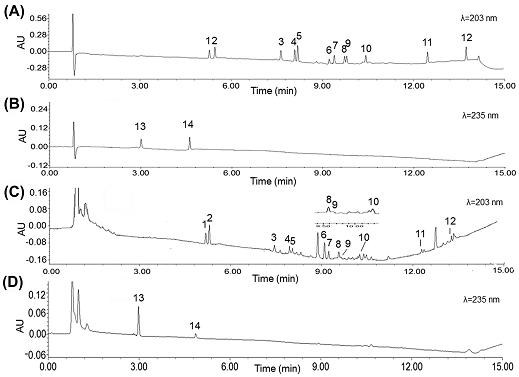

Supplement: Supplementary file 1 [file DataSheet_1.zip › the supplementary materials/Fig. S2.tif]

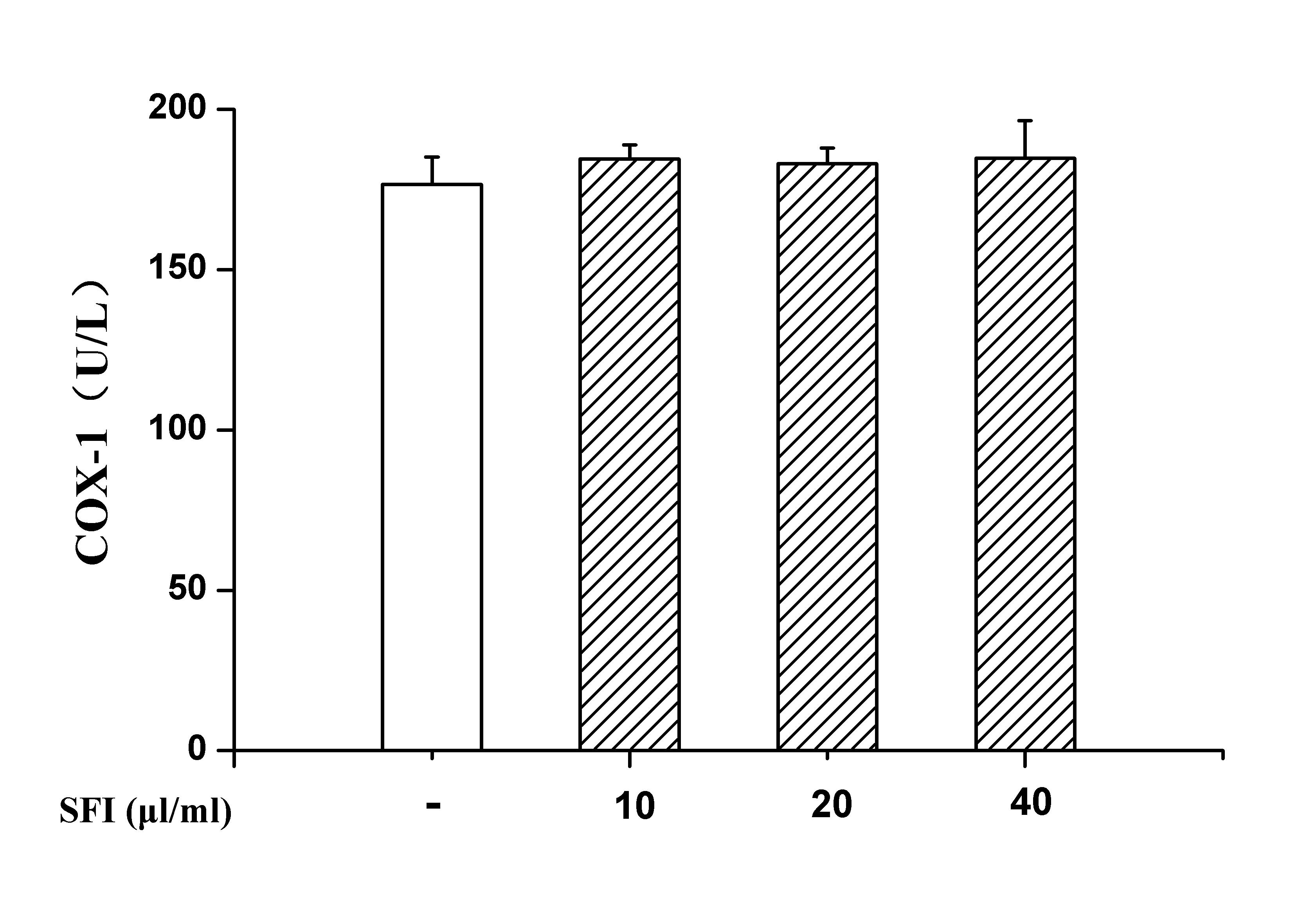

Supplement: Supplementary file 1 [file DataSheet_1.zip › the supplementary materials/Fig. S3.tif]

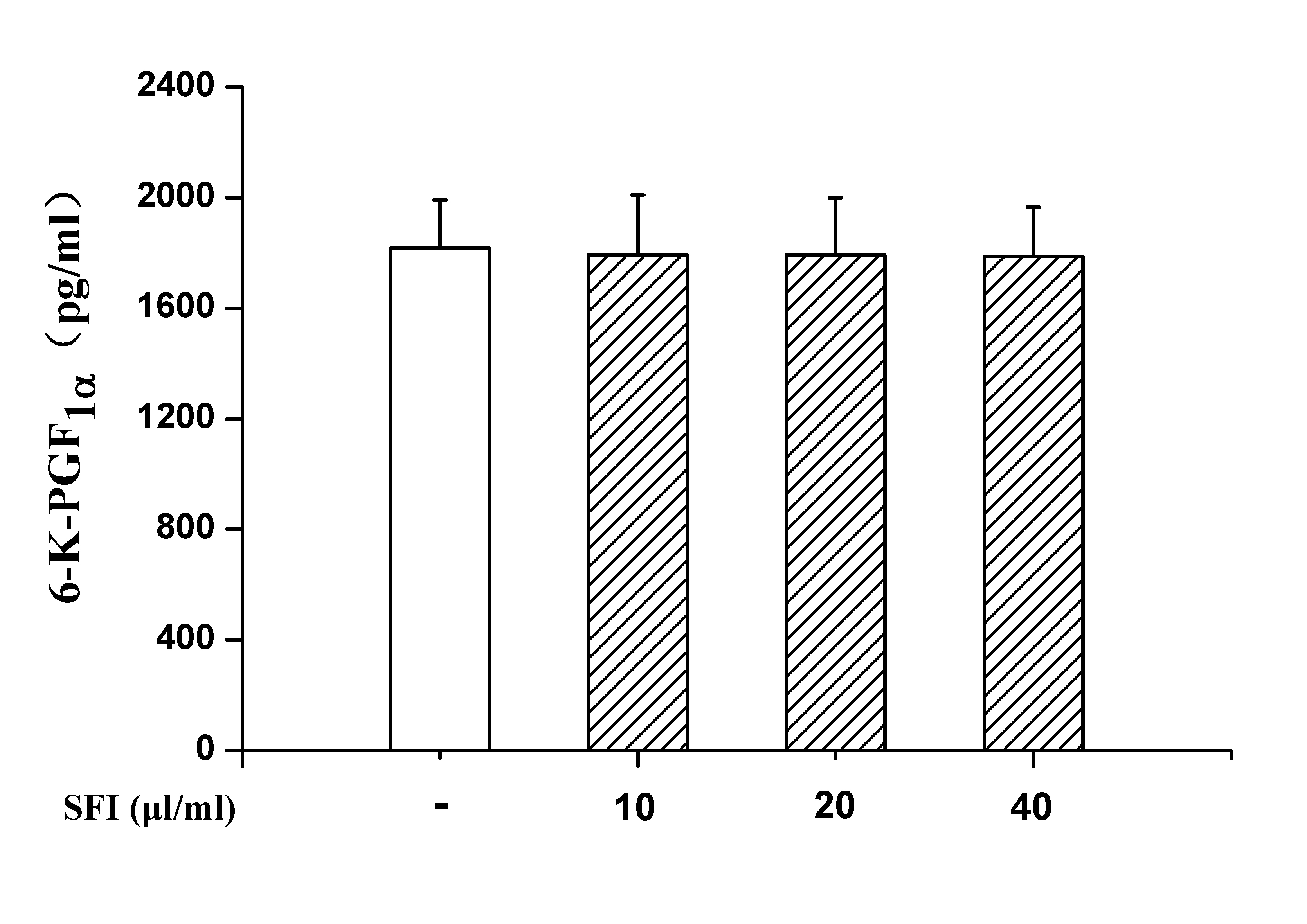

Supplement: Supplementary file 1 [file DataSheet_1.zip › the supplementary materials/Fig. S4.tif]

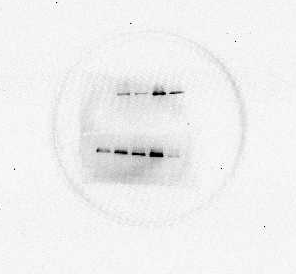

Supplement: Supplementary file 2 [file DataSheet_2.zip › original image/eNOS Ser1177 ( original image).tif]

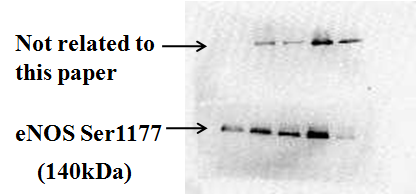

Supplement: Supplementary file 2 [file DataSheet_2.zip › original image/eNOS Ser1177 (marked).tif]

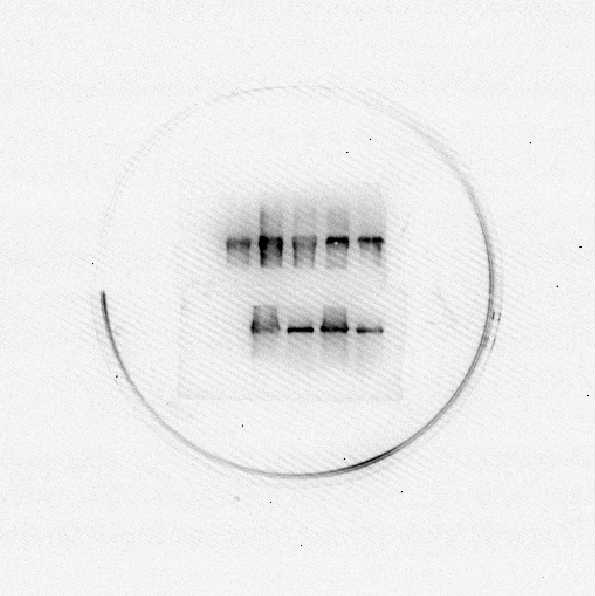

Supplement: Supplementary file 2 [file DataSheet_2.zip › original image/eNOS Thr495 ( original image).tif]

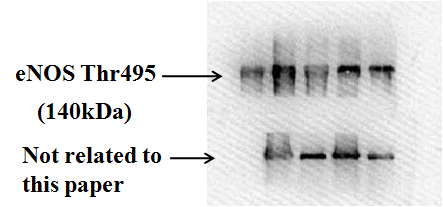

Supplement: Supplementary file 2 [file DataSheet_2.zip › original image/eNOS Thr495 (marked).tif]

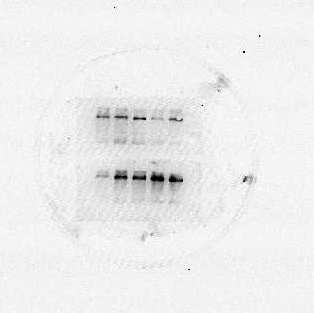

Supplement: Supplementary file 2 [file DataSheet_2.zip › original image/total eNOS ( original image).tif]

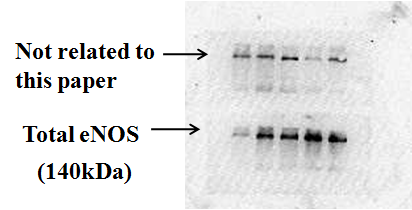

Supplement: Supplementary file 2 [file DataSheet_2.zip › original image/total eNOS (marked).tif]

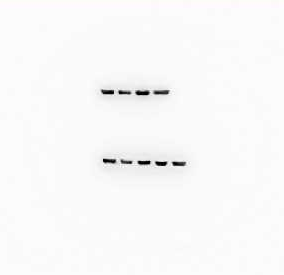

Supplement: Supplementary file 2 [file DataSheet_2.zip › original image/a┬-actin ( original image).tif]

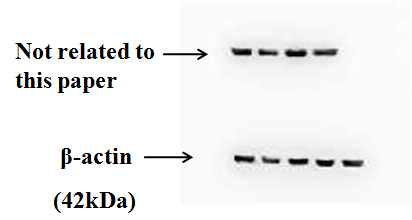

Supplement: Supplementary file 2 [file DataSheet_2.zip › original image/a┬-actin ú¿marked).tif]
