# Supplementary material for: Shenfu Injection Promotes Vasodilation by Enhancing eNOS Activity Through the PI3K/Akt Signaling Pathway In Vitro
Source: Front Pharmacol. 2020 Feb 26;11:121. doi: 10.3389/fphar.2020.00121 (PMC7054240; doi:10.3389/fphar.2020.00121)
Supplement: Supplementary file 3 [file DataSheet_3.docx]

**

Fig. 1 Chemical structures of Ginsenoside and Aconitine (Wu et al., 2016)

Fig. 2 Representative chromatograms for simultaneous quantification of the 14 active compounds in Shenfu injection: (A) mixed standards at 203 nm; (B) mixed standards at 235 nm; (C) Shenfu injection sample at 203 nm; and (D) Shenfu injection sample at 235 nm. Peak 1 represents Re, peak 2 Rg_1_, peak 3 Rf, peak 4 S-Rg_2_, peak 5 S-Rh_1_, peak 6 Rb_1_, peak 7 Rc, peak 8 Rb_2_,peak 9 Rb_3_, peak 10 Rd, peak 11 S-Rg_3_, peak 12 S-Rh_2_, peak 13 benzoylmesaconine, and peak 14 benzoylhypacoitine (A. H. Ge et al., 2015).
